# The -omics Era- Toward a Systems-Level Understanding of *Streptomyces*

**DOI:** 10.2174/138920211797248556

**Published:** 2011-09

**Authors:** Zhan Zhou, Jianying Gu, Yi-Ling Du, Yong-Quan Li, Yufeng Wang

**Affiliations:** 1 College of Life Sciences, Zhejiang University, Hangzhou 310058, P.R. China; 2 Department of Biology, University of Texas at San Antonio, San Antonio, TX 78249, USA; 3 South Texas Center for Emerging Infectious Diseases, University of Texas at San Antonio, San Antonio, TX 78249, USA; 4 Department of Biology, College of Staten Island, City University of New York, Staten Island, NY 10314, USA

**Keywords:** *Streptomyces*, genomics, systems biology, transcriptomics, proteomics, metabolomics, interactomics, antibiotics, bioinformatics.

## Abstract

*Streptomyces* is a group of soil bacteria of medicinal, economic, ecological, and industrial importance. It is renowned for its complex biology in gene regulation, antibiotic production, morphological differentiation, and stress response. In this review, we provide an overview of the recent advances in *Streptomyces* biology inspired by -omics based high throughput technologies. In this post-genomic era, vast amounts of data have been integrated to provide significant new insights into the fundamental mechanisms of system control and regulation dynamics of *Streptomyces*.

## INTRODUCTION

*Streptomyces* is a group of Gram-positive soil-dwelling bacteria. It forms the largest genus in the *Actinobacteria*, which includes over 580 species [1]. Streptomycetes have received much research attention due to:

1. their medicinal and pharmaceutical significance. Streptomycetes produce over half of naturally-occurring or naturally-derived antibiotics [2], and many other bioactive compounds ranging from anticancer agents, antiparasitic drugs, antifungals, immunosuppressants to herbicidal agents [3]. They are the products of complex secondary metabolic pathways. For example, actinomycin was the first antibiotic isolated from *Streptomyces* [4]; it is an inhibitor for DNA transcription and has been used in chemotherapy for cancer treatment [5]. Streptomycin was first isolated from *S. griseus* in 1943; it is an inhibitor for protein synthesis and has been used as a broad spectrum antibiotic, treating tuberculosis and *Yersinia pestis*, for example [6, 7]. Recently, we isolated a novel strain, *S. chattanoogensis* L10, which is capable of producing natamycin, a class of polyenes macrolide antibiotics that can block fungal growth [8].
2. their contribution to the carbon recycling process. Streptomycetes are ubiquitous in soil and play a crucial role in global carbon cycle by degrading the insoluble remains of other organisms, including lignocellulose and chitin, which are otherwise hard to degrade.
3. their distinct developmental biology involving sophisticated morphological differentiation. Unlike traditional bacteria, streptomycetes have a life cycle that includes transitions between differentiated “tissues”. The life cycle starts with a resting spore, which germinates under certain conditions to form a vegetative or substrate mycelium. Branching hyphae of the vegetative mycelium grow and extend into the substrate to reach nutrients. When food is scarce or in response to other stimuli, some branches start growing into the air, becoming aerial mycelium. Finally, the distal parts of aerial hyphae yield spores after the elongation and partition process. *Streptomyces* serves as a model system for studying the molecular mechanisms underlying microbial differentiation, as the morphological differentiation process involves orchestrated regulation of cell growth, cell-cycle progression, cell wall assembly, sporulation, extracellular signaling and the interplay with a prodigious secondary metabolism [9].
4. their unusually complex gene regulatory networks. The adaptation of streptomycetes to a continuously changing soil environment requires the bacteria to rapidly respond to diverse environmental and physiological signals and stress conditions, such as changes in environmental temperature, pH, humidity, starvation, and radiation, etc. In *S. coelicolor* A3(2), 65 RNA polymerase sigma factors may act as the central players in the gene regulatory networks [2]; they are regulated by specific anti- and anti-anti-sigma factors, forming gene clusters on the chromosome. A large number of regulatory proteins have been identified in the genome of *S. coelicolor* A3(2), which can be pathway-specific, pleiotropic, or global [10, 11].
5. their exceptionally powerful secretion systems. Streptomycetes secrete a huge number of proteins through at least three secretion systems: (a) the general secretary (Sec) pathway, which exports unfolded proteins across the cytoplasmic membrane. (b) the ESX-1/type VII (Esx) secretion system, which secretes small proteins and other proteins without any detectable signal peptide, and (c) the twin-arginine translocation (Tat) pathway, which transports prefolded proteins and a group of hydrolytic enzymes [12]. These systems facilitate nutrient acquisition, for example, by obtaining the breakdown products from insoluble nutrient sources such as chitin and cellulose. They also secrete peptidases and their associated inhibitors which are believed to be integral regulators of morphological development in streptomycetes. Streptomycetes also contain a remarkable number of ABC transporters (~81 typical permeases in *S. coelicolor* A3(2)), some of which may facilitate the transport of secondary metabolites, including antibiotics. Moreover, streptomycetes have been used recently in the biotechnology industry as a host system for heterogeneous expression of proteins, offering better yields and simpler downstream processing [13, 14].
6. their potential use in the study of pathogenesis and virulence. The majority of the species of *Streptomyces* are non-pathogenic; only a fraction causes diseases. *S. somaliensis* and *S. sudanensis* can cause actinomycosis in humans, a chronic disease that commonly affects the face and neck [15, 16]. About a dozen species are plant pathogenic [17]. *S. scabiei*, *S. turgidiscabies* and *S. acidiscabies* are the causative agents for potato scab, a disease that is responsible for significant economic losses, and is characterized by the lesions on tuber surfaces [18, 19].

In summary, streptomycetes represent a group of bacteria of medicinal, pharmacological, ecological, industrial, economic and pathological importance. The aforementioned key characteristics of the streptomycetes are a prime example of the enormous evolutionary changes that can occur during a dynamic life cycle involving morphological differentiation, nutrient competitions, and stress responses. *Streptomyces* is an ideal model system to study how the bacteria develop the genetic variability that is associated with their response to environmental challenges and other adaptive phenotypes. Excellent surveys on *Streptomyces* evolution and genetics exist in the literature, offering indepth reviews on the frontier of this important field [12, 20]. The completion of multiple *Streptomyces* genomes, rapid advances in deep sequencing technology, and subsequent studies on transcriptomes, proteomes, metabolomes, interactomes, and secretomes make this an appropriate time to survey -omics-centered developments in *Streptomyces* biology. 

## GENOMICS OF *STREPTOMYCES*

Genomics of *Streptomyces* is coming of age after 50 years of genetic research since 1950s [3, 20]. The complete genome sequences of *S. coelicolor* A3(2) and *S. avermitilis* were published in 2002 and 2003, respectively [2, 21]. To date genome sequence data from 32 strains of *Streptomyces* are in various stages of completion and are available to the research community in public databases (http://www.ncbi. nlm.nih.gov/genomes/lproks.cgi): nine strains with complete sequences and 23 with whole genome shotgun draft sequences (Table **1**). Genome sequencing of a number of other streptomycetes is in progress, but the information on various industrial stains with complete sequences is not publically available due to proprietary protection. 

Each of the nine completed *Streptomyces* species has its unique properties and biological/medicinal importance: *S. coelicolor* is the best studied *streptomycetes* and it can produce various antibiotics, including actinorhodin, methylenomycin, undecylprodigiosin, and CDA (calcium-dependent antibiotic) [22]; *S. avermitilis* [21, 23], *S. griseus* [24], *S. bingchenggensis* [25] and *S. sp.* Tü6071 [26] are industrial production bacteria, which produce commercially important antibiotics - avermectin, streptomycin, milbemycin, and phenalinolactones, respectively; *S. scabiei* [27] is a plant pathogen that causes potato scabs, a disease of economic significance; *S. cf. griseus* (XylebKG-1) [28] is the first strain of *S. griseus* that was reported to be associated with an insect (the ambrosia beetle *Xyleborinus saxesenii*), which provides a model system for the study of bacteria-insect interactions; *S. flavogriseus* possesses hemicellulose debranching activities required for extensive degradation of cellulose and xylan [29]; *S. venezuelae* is unique in its complex process of morphological development as it sporulates to near completion in liquid medium, unlike other streptomycetes which differentiate only on solid medium.

### Commons Features in the Sequenced *Streptomyces* Genomes

**Fig. (1):**
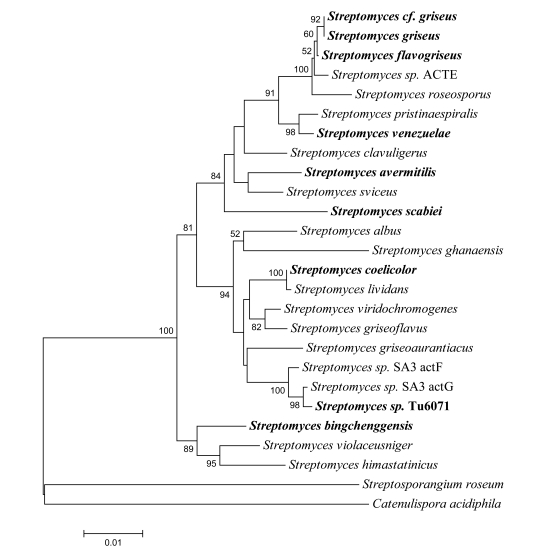
Phylogenetic tree of 16S ribosomal RNA of genome sequenced *Streptomyces*. The tree was constructed using the neighbor-joining method [100]. *Streptosporangium roseum* and *Catenulispora acidiphila* were set as outgroups. The names of nine *Streptomyces* stains, which have complete genome sequences, are shown in the bold type. The Maximum Parsimony and Maximum Likelihood methods give virtually the same topology (data not shown).

**Fig. (2):**
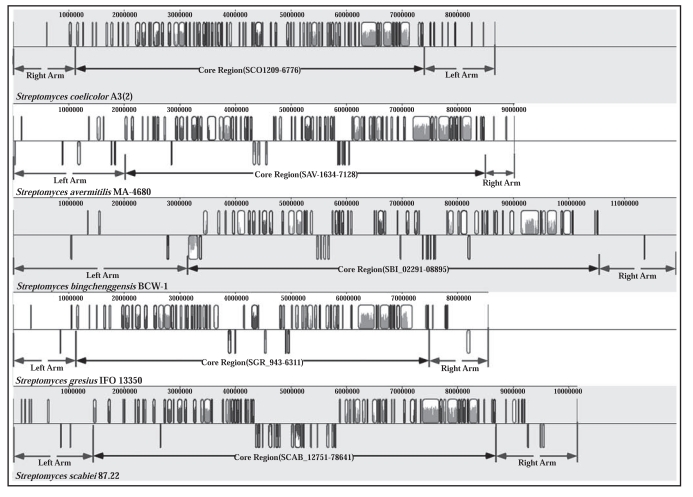
Genome alignment of five *Streptomyces* genomes, using MAUVE 2.3.1 [45]. The core region and the arm regions are marked in each chromosome. Core region: SCO1209-6776; SAV_1634-7128; SBI_02291-08895; SGR_943-6311; SCAB_12751-78641. The reverse complement of the *S. coelicolor* sequence was used for this analysis.

Despite the diverse physiological features and ecological niches of the streptomycetes, their genomes are relatively conserved, consistent with the phylogenetic analysis of 16s rRNA sequences (Fig. **1**). The common features include:

1. The chromosomes are linear, GC-rich (about 70%), and of large size ranging from 6.5 Mbp to 11.9 Mbp (Table **1**). The linearity of chromosome is a distinct property of *Streptomyces*, whereas the majority of bacteria and archaea possess circular chromosomes. The linear chromosome in streptomycetes is highly unstable and undergoes large rearrangements including massive DNA deletions and amplifications. Several genes (*tpg*, *tap*, and *ttr*) have been shown to be related to chromosomal linearity [30-32]. Although it remains unclear whether linearity or circularity is the ancestral form of the bacterial chromosome [33, 34], it is believed that linearity may confer evolutionary advantages: (a) for sustaining of larger chromosomal size. As shown in the whole genome alignment (Fig. **2**), the central core region on the chromosome is highly conserved and syntenous, which includes the genes that are essential for replication, transcription, translation and cell division, while the terminal regions of the chromosome often contain lineage-specific genes. This modular organization may allow the increase of chromosome size from the telomeric regions without causing functional disruption in the core region. (b) for the genetic instability that accounts for important pleiotropic traits in *Streptomyces*, including morphological differentiation, secretion of extracellular enzymes, and particularly, the rich repertoire of secondary metabolism [35], many of which are controlled or regulated by the genes that are located in the two arm regions. The high G+C content of streptomycetes is likely generated by mutational biases [36]. Such GC-biased mutational pressure determines the nonrandom codon usage. For example, in *S. venezuelae*, codon AAA (for Lys) is only used in 2.2% of the occurrences [37]. Codon usage bias can be viewed as an evolutionary strategy for translational optimization. It seems to be, particularly in streptomycetes, an effective mechanism for regulation of gene expression. For example, in *S. coelicolor* A3(2), the *bldA* gene encodes the only tRNA species capable of reading the rare Leu codon UAA efficiently [38]; this codon is found in only a few genes that are implicated in morphological differentiation and secondary metabolism [39, 40]. An alternative hypothesis of evolutionary advantages for a higher G+C genome is that the GC bias is associated with enhanced thermal stability of the dsDNA topology and protein structures [41].
2. Numerous gene clusters for complex regulatory systems are present in the *Streptomyces* genomes. For example, about 12% of the open reading frames (ORFs) in *S. coelicolor* A3(2) were predicted to play regulatory roles, including at least 53 sensor kinases-response regulator pairs that constitute the two-component regulatory systems [2, 42]. An extraordinary high number of duplicated genes are present in the genome and may function in a ‘tissue-specific’ manner, in response to different stimuli or operating in different developmental stages [2, 43]. Sixty-five and 60 putative sigma factors were reported in the genomes of *S. coelicolor* A3(2) and *S. avermitilis*, respectively, including 45 and 47 in the extracytoplasmic function (ECF) subfamily, which are implicated in the coordination of transcriptional regulation in response to various environmental conditions [2, 21, 44].
3. Abundant genes are associated with secondary metabolism. Prior to the release of the *S. coelicolor* A3(2) genome, little was known about the molecular mechanisms underlying secondary metabolism, nor the regulators. It was exciting that 18 additional clusters of genes were identified in *S. coelicolor* A3(2), which encode the enzymes that catalyze the synthesis of novel secondary metabolites such as coelichelin, coelibactin, eicosapentaenoic aid, hapanoid, and calcium-dependent antibiotic [2]. Moreover, the *S. griseus* genome harbors 30 new gene clusters for secondary metabolism, which are predicted to be associated with the biosynthesis of carotenoid, hopanoid, and terpenoid, to name a few [24]. At least 23 biosynthetic clusters were reported in *S. bingchenggensis*, including clusters for novel compounds such as milbemycin and bingchamide [25]. It is noteworthy that many of these gene clusters are located in the distal subtelomeric regions of the chromosome.

### Comparative Analysis of the *Streptomyces* Genomes

At the time of writing, among the nine completed *Streptomyces* genomes, only five have been fully annotated. Some streptomycetes carry plasmid(s): *S. coelicolor* A3(2) has a linear plasmid SCP1 and a circular plasmid SCP2, and *S. avermitilis* has a linear plasmid SAP1. Our comprehensive analysis of these five genomes revealed that their linear chromosomes can be divided into three regions, the central core region and the two arm regions with clearly defined boundaries based on the genome alignment using the Mauve program version 2.3.1 [2, 24, 33, 45] (Fig. **2**). The core regions of the five chromosomes are: SCO1209-6776, SAV_1634-7128, SBI_02291-08895, SGR_943-6311, and SCAB_12751-78641. Terminal inversed repeats (TIRs) are present at the ends of all the chromosomes and linear plasmids [46]. The lengths of TIRs vary significantly, ranging from 138 bp (*S. bingchenggensis*) to 133 kb (*S. griseus*). Notably, the TIRs in the chromosome of *S. avermitilis* are only 167 bp long, even shorter than the TIR in its linear plasmid SAP1 which are 434 bp long. A number of terminal proteins (TPs) which are covalently bound to the 5' end of the telomeric DNA have been identified and characterized, suggesting that TPs are essential for the replication of linear chromosome and linear plasmids in streptomycetes [31, 47].

We further identified core genome components that are shared by all the five genomes and the genes that have undergone lineage-specific expansions (LSEs) [48]. The level of sequence conservation in the core region is generally much higher than that in the arms which tend to harbor lineage-specific genes [34]: about 43.8%-53.7% of the genes in the core region are commonly present in all the five genomes, while a significantly lower proportion (11.0%-21.6%) is present in the arm regions, and the lowest proportion (5.9%-12.3%) is found in the plasmids. *S. coelicolor*, *S. avermitilis* and *S. griseus* have similar chromosome sizes (8.7 Mb, 9.0 Mb and 8.5 Mb, respectively), and also have similar proportions of core genome components in their core region (~53%). With increasing chromosome size, the proportion of the core genome elements becomes lower, as shown in the genomes of *S. scabiei* (45.5%) and *S. bingchenggensis* (43.8%). The lower proportion of the core genome components in the arm regions suggests that the terminal regions might be the hot spots for the gene loss or acquisition events *via* lateral gene transfer. 

**Fig. (3):**
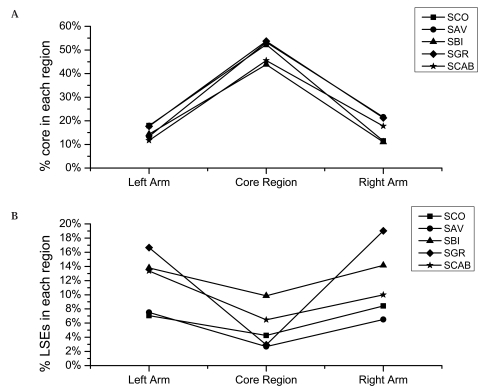
Genome topology of five *Streptomyces* species. (**A**), Proportion of genes that are shared by all the five *Streptomyces* genomes (Core genome) in three separated regions of each chromosome. (**B**), Proportion of genes that have undergone lineage specific expansion (LSE) in three separated regions of the chromosome. SCO: the chromosome of *S. coelicolor*; SAV: the chromosome of *S. avermitilis*; SBI: the chromosome of *S. bingchenggensis*; SGR: the chromosome of *S. griseus*; SCAB: the chromosome of *S. scabiei.*

LSEs are abundant, accounting for about 6.5%-19.0% and 12.5%-43.6% of the genes in the arm regions and in the plasmids, respectively (Fig. **3**). By contrast, merely 2.7%-9.9% of the genes in the core region are lineage-specific expansions. An extreme case is observed in SCP1, a giant plasmid in *S. coelicolor* A3(2), which contains 43.6% LSE genes (153/351). Moreover, all except one of the 153 LSE genes are located in the TIR. For *S. griseus* which has the longest TIRs, the proportions of LSE genes in the arm regions (16.6% and 19.0%) are the largest among the five genomes. *S. bingchenggensis* which has a much larger genome (11.9 Mb) than the other four species and, unsurprisingly, has the largest number and proportion of LSE genes (14.2% and 13.9% in two respective arms, and 9.9% in the core region). *S. coelicolor* A3(2) and *S. avermitilis* have relatively lower proportions of LSE genes: the former contains 7.1% and 8.4% LSEs in the arm regions, and 4.2% in the core region, and the latter has 7.5% and 6.5% in the arm and 2.7% in the core. 

It is well known that the telomeric ends of the chromosome of streptomycetes are highly dynamic. A large portion of terminal proteins (60.0%-83.3%) are lineage-specific expansions. For example, in *S. coelicolor *A3(2)*, *the 21.7 kb long TIRs in the chromosome contain 20 duplicated genes, 60.0% of which are LSEs [48], and the TIRs in its linear plasmid SCP1 contain 76 duplicated genes, 73.7% of which are LSE. The TIRs are believed to play an important role in the genomic plasticity of streptomycetes by offering increased flexibility for homologous recombination, transposition, and other chromosomal rearrangement events [49]. Transposable elements were postulated to be major agents of the genetic instability in the terminal ends, evidenced by the traces of abundant intact or partial sequences that code for transposases and integrases. The lengths of transposase and integrase genes vary significantly in the five genomes, suggesting their heterogeneous sources, activities, and diverse routes of adaptation. Undoubtedly, lateral gene transfer and integrase- or transpose-dependent gene acquisition play an important role in functional divergence and genome evolution in streptomycetes. 

Comparative analysis of these five *Streptomyces* genomes also confirmed the presence in all five genomes of 120 of the previously defined signature proteins for *Actinobacteria* (Supplementary Table **1**), which include 28 signature proteins specific for all the *Actinobacteria* surveyed by Gao *et al.* [50], 56 signature proteins for specific Actinobacterial subgroups and 36 *Actinobacteria*-specific proteins with a sporadic distribution pattern [33, 50]. Seventy of these 120 signature proteins are annotated as hypothetical proteins with no discernible functionality. The functionalities for the remaining signature proteins range from transcriptional regulation, protease activity, to transport activity. A large number of signature proteins are membrane proteins. 

## THE POST-GENOMIC ERA AND SYSTEMS BIOLOGY

The *Streptomyces* research community is rapidly advancing upon the post-genomic era in which high volumes of genomic data are integrated with the results from emerging high throughput experimental technologies to elucidate the structure and dynamics of biological systems. Technologies such as next-generation sequencing [51], DNA chip, ChIP-chip (chromatin immunoprecipitation-on-chip), mass-spectrometry, two-hybrid analysis [52] have enabled the rapid and large-scale characterization and profiling of the primary sequences, expression, regulation and action of the genes at the level of the transcriptome, proteome, metabolome, and interactome. 

### Transcriptomics and Cistromics

The release of genome data made it possible to carry out global expression studies. The first transciptomic analysis of *Streptomyces*, coupling microarray chip technology and mutational analysis, was focused on the delineation of genes/regulators/regulons that are involved in the transition of growth phases from primary to secondary metabolism [53]. The array chip included the 50- to 2400-bp long PCR amplified fragments corresponding to the putative ORFs in *S. coelicolor.* A hierarchical clustering and a machine learning algorithm showed that about 22% (1100/4960) of the genes in *S. coelicolor* exhibited differential expression through the various growth phases from vegetative growth, to the primary to secondary transition stage, and to the post-transitional stage. Combining the chromosomal positional analysis with time-series classification, the authors identified co-regulated clusters of contiguous genes which may play a role in pathways for the production of the pigmented antibiotics undecylprodigiosin (Red) and actinorhodin (Act).

Microarray analysis has become one of the most powerful approaches to the study of gene regulatory networks in *Streptomyces.* Currently, three types of custom made microarray chips are available to the research community from the *Streptomyces coelicolor* Microarray Resource (http://www.surrey.ac.uk/fhms/microarrays/): (1) the spotted oligoarray which includes the 50mers corresponding to the ORFs in the chromosome and the plasmid SCP1. This chip is specifically designed for *S. coelicolor* or its close evolutionary relatives; (2) the spotted PCR-product array which includes the probes (150-500 bp long) amplified from PCR assays. This chip can be used for the transcriptional profiling of streptomycetes that are remote relatives of *S. coelicolor*; (3) the ChIP-on-chip array which includes 44,000 experimentally validated 60mers that are manufactured using the cutting edge ink-jet in situ synthesis (IJISS) technology. This chip coupled with chromatin immunoprecipitation can be used to identify and characterize the cistrome, the whole complement of DNA-binding sites for target proteins [54]. A wide array of intriguing questions in *Streptomyces* have now been investigated, ranging from the delineation of whole genome operon maps [55, 56] to the identification of specific gene regulatory networks, including:

1. How do sigma factors and other pleiotropic regulators control global transcription? Sigma factors are master regulators in *streptomycetes* that coordinate their response to environmental and physiological cues. Microarray chip serves as an ideal platform to investigate the role of sigma factors which involve cascades of regulators and effectors. Lee *et al.* [57] explored the gene regulatory networks controlled by σ^B^, a central regulator for osmotic stress response. Two hundred and eighty (280) genes were found to be induced by 0.2M KCI, which causes osmotic shock of *S. coelicolor*, including those coding for two paralogous sigma factors of σ^B^ (σ^L^ and σ^M^), σ^hrdD^, RsbV (an anti-anti-sigma factor for σ^B^), catalases, bacterioferritin, gas vesicle components, sulphur assimilation, and many anti-oxidative proteins. The disruption mutant phenotypes suggest that σ^B^, σ^L^ and σ^M^ function in a sequential manner in morphological differentiation to prevent osmotic damages: σ^B^ in formation of aerial mycelium, σ^L^ in spore formation forming spores and σ^M^ for efficient sporulation. Similarly, Lian *et al.* [58] investigated the transcriptomic profiles of an AfsS disruption mutant in *S. coelicolor.* AfsS is a small pleiotropic regulator that functions like sigma factors. The mutant completely lost its ability to produce actinorhodin. Microarray and quantitative PCR analyses revealed that genes showing significant differential expression were associated with antibiotic production, phosphate starvation response, nitrogen metabolism, and sulfate assimilation. The observation that most of these genes were down-regulated in the mutant suggested a role for AfsS as a positive regulator.
2. How do bacterial hormones regulate the production of antibiotics? A number of γ-butyrolactone signaling molecules such as A-factor (2-isocapryloyl-3R-hydroxymethylg-butyrolactone) [59], VB (virginiae butanolides) [60], and SCB1 [61], which are key regulators for the onset of antibiotic biosynthesis, have been identified in streptomycetes. Microarray analysis revealed that a hypothetical type I polyketide biosynthetic cluster of genes might be directly repressed by ScbR, a regulator for γ-butyrolactone SCB1 binding [62]. This cluster includes two SARP (*Streptomyces* antibiotic regulatory protein) homologs (KasO and SCO6288), and SCO6286, a homologue of ScbR. Promoter analysis using gel retardation and DNase I footprinting suggested that KasO is directly regulated by ScbR. DNA microarray experiments have also been used to determine the gene clusters which are direct or indirect targets of AdpA, a transcriptional activator that plays a pivotal role in A-factor regulatory networks for secondary metabolism and morphogenesis in *S. griseus* [24]. Using comparative transcriptomic analysis, Kang *et al.* [63] identified a novel putative transcription factor WblA in *S. peucetius*, a clinically important strain that produces the anticancer agent Doxorubicin. WblA was shown to be a down-regulator of doxorubicin biosynthesis in *S. peucetius*, and a pleiotropic regulator for secondary metabolism and morphological differentiation in *S. coelicolor.* In addition, SCO1712, a TetR family transcriptional regulator gene, was found to negatively regulate antibiotic production in *S. coelicolor* [64]. A subsequent microarray analysis of a *S. coelicolor* Δ*wblA* ΔSCO1712 double knockout mutant revealed 28 WblA- and SCO1712-dependent genes including multiple genes that are implicated in antibiotic synthesis pathways, membrane proteins and regulatory proteins. Fourteen (14) genes were found to have no significant changes in their expression levels, suggesting their independence of WblA or SCO1712. This group included a carbon flux-regulating gene SCO5426, whose disruption in the Δ*wblA* ΔSCO1712 mutant background stimulated the production of actinorhodin, suggesting a synergistic strategy of coupling regulatory circuits and precursor flux [65]. The role of AveI, a negative regulator for avermectin biosynthesis has also been interrogated in *S. avermitilis* using NimbleGen microarrays [66].
3. How do signal transduction pathways control morphogenesis? The identification of a number of important mutants began to unveil the molecular mechanisms underlying the complex developmental and growth cycle of streptomycetes: *bld* mutants lack the ability to produce aerial hyphae [67], and *whi* mutants produce aerial hyphae but fail to produce mature spores [68]. Microarray analyses have enabled the identification of novel components of morphogenesis, representing at least two independent developmental pathways: (a) the chaplin pathway. A family of chaplins (*S. coelicolor* hydrophobic aerial proteins) (ChpA-H) were found to be down-regulated in a *bldN* mutant that was unable to erect aerial hyphae. Chaplins are a group of hydrophobic secreted proteins required for the growth of aerial hyphae on both minimal and rich medium [69]; (b) the *rag* (RamR-activated genes) pathway. Extracellular complementation assays suggested that the *bld* gene is directly or indirectly involved in a signaling cascade that releases morphogenetic molecules such as SapB, a biosurfactant that reduces the surface tension at the water-air-colony interface, thereby enabling the upright growth of nascent aerial hyphae [70, 71]. SapB was found to be activated by a key regulator RamR [72]. Microarray analysis of RamR deletion mutants and overexpression mutants identified a novel cluster of *rag* that can potentially modulate a SapB independent signaling pathway that is important for aerial hyphae formation and sporulation [73]. The *rag* cluster includes two subunits of an ABC transporter, a putative histidine kinase, and a RamR paralog. S1 nuclease mapping confirmed that the *rag* cluster may be a direct target for RamR binding. Recently, ChIP-chip has been introduced to the morphogenesis research to establish the DNA-protein binding interaction maps in streptomycetes. A huge cluster of BldD regulon was identified by ChIP-chip, comprising 167 genes, of which 42 encode DNA-binding proteins. Many of the BldD target genes were shown to be important regulators of morphological development, including the proteins in the *bld* and *whi* families, and of antibiotic production, suggesting that BldD is a master regulatory protein [74].
4. How is the stress response orchestrated? The stress response is one of the most important adaptive characteristics for streptomycetes that live in the changing soil environment. The genome annotation predicted many genes related to stress responses. Microarray technology has been used to elucidate the components of stress response networks. Bucca *et al.* [54, 75] performed transcriptomic analyses of the heat shock protein (hsp) chaperone network in *S. coelicolor.* HspR is a negative regulator in the *dnaK* operon. Comparative transcriptomic analysis between wild-type and the *hspR*-disruption mutant identified a cluster of target genes modulated by HspR: *dnaK*, *clpB* ATPase-SCO3660 operons and *lon* protease, which form the HspR regulon. In addition, HspR was shown to interact with rRNA (*rrnD* operon) and specific tRNA genes (tRNAGln/tRNAGlu), in response to the higher demand for Gln and Glu induced by heat shock. Numerous studies using microarray were focused on the bacteria response to various starvation conditions. Hesketh *et al.* [76] explored the gene regulatory network in response to amino acid starvation involving a stringent factor ppGpp [77]. Affymetrix chips were used to assess the transcriptional profiles in a null mutant for *relA*, which encodes ppGpp synthetase. A large number of genes were repressed by ppGpp synthesis, including those are related to carbon metabolism, cell wall biosynthesis, ATP synthesis, fatty acid biosynthesis, and purine/pyrimidine biosynthesis. Other genes whose expression were affected by ppGpp production included transporters and a cluster of conservons (cvns), the conserved operons present in *Streptomyces* which contain genes encoding a putative sensor histidine kinase and an ATP/GTP-binding protein. More importantly, ppGpp synthesis repressed the transcription of the major vegetative sigma factor σ^hrdB^ and induced an alternative ECF sigma factor SCO4005. It also triggered the transcription of genes associated with secondary metabolism and alternative ribosomal protein synthesis. Transcriptomic and cistromic studies on stress responses have been extended to *S. venezuelae*, a model system for *Streptomyces* physiology and developmental biology [78]. The GlnR regulon plays important roles in nitrogen metabolism. ChIP-chip analysis identified 36 GlnR binding sites in the genes that are associated with nitrogen metabolism, secondary metabolism, the synthesis of catabolic enzymes and transport activities.

## PROTEOMICS

Complementary to transcriptomic analysis, a proteomic level of understanding enables researchers to put genomic information in context to determine the function of expressed proteins. The characterization of a proteome is typically composed of the following steps: firstly, protein isolation; secondly, separation and visualization of individual proteins in the mixture, for example, by two-dimensional (2-D) gel electrophoresis; thirdly, quantitative analysis of the proteins by mass spectrometry or N-terminal sequencing of individual proteins. The development of high throughput technologies such as matrix-assisted laser desorption ionization-time-of-flight (MALDI–TOF) mass spectrometry [79], capillary liquid chromatography, followed by tandem mass spectrometry (LC/MS/MS) [80], and Multidimensional Protein Identification Technology (MUDPIT) which couples liquid chromatography to tandem mass spectrometry [81], have allowed system-wide characterizations of proteomes [82]. 

The complex life cycle of streptomycetes requires developmental switches that are likely to be reflected in the tempo-spatial-specific expression of proteins. Proteomic analyses have revealed previously uncharacterized components and dynamics in the cellular networks in streptomycetes. A first glimpse of the proteome in *S. coelicolor* showed that the proteins associated with primary and secondary metabolism were abundant, and more significantly, post-translational modification (PTM) appeared to be a common regulatory mechanism for *Streptomyces* proteins [83]. 

The secretome, the complement of secreted proteins, has been systematically defined and characterized in streptomycetes [12]. Using 2-D gel and MALDI-TOF, Kim *et al.* [84] confirmed that streptomycetes secrete a large number of proteins. Several unexpected proteins were found in the extracullar fraction. For example, a putative formyltransferase (SCO0499), which was predicted to be related to the production of the siderophore coelichelin was found in the secretome. Further extracellular proteomic analysis of a null mutant for *bldA* gene, a critical regulator for antibiotic production and morphological differentiation identified a *bldA*-dependent target gene, SCO0762, which encodes a putative serine-protease inhibitor, suggesting the role of an extracellular protease cascade in differentiation. This cascade is likely regulated by a pleiotropic regulator AdpA. Similarly, the important role of AdpA in morphological development for *S. griseus* was evidenced by the observation of 38 AdpA-dependent secreted proteins including proteases, glycosyl hydrolases and esterases, in a combined transcriptomic and proteomic analysis [85]. Proteomics has also shed light on the sophisticated transport mechanism in streptomycetes. The twin-arginine translocation (Tat) pathway was postulated to be a major transport system that is dedicated to the translocation of prefolded proteins across membranes [86]. Substrates of the Tat pathway contain cleavable N-terminal signal peptides with a highly conserved double arginine motif. Forty-three (43) secreted proteins were found to be potentially Tat-dependent *via* 2-D gel and MUDPIT proteomic assays, including 25 proteins that contain Tat-targeting signal peptides verified by a Tat reporter assay. These Tat target proteins were predicted to be involved in phosphate acquisition, carbohydrate metabolism, lipid metabolism, and secondary metabolism [87]. A subsequent study in the causative pathogen of potato scab *S. scabies* that used the comparative extracellular proteomics and a Tat transport reporter validation assay [88] identified 47 Tat-dependent substrates. Seven of these Tat-substrates could be potential virulence factors, as indicated by a reduced virulence phenotype in the gene disruption mutants. This study provided solid evidence for the involvement of the Tat export pathway in pathogenesis, and it may serve as a target for novel drug development [89]. In addition to the study of transport systems, the proteomic approach has also been applied to the delineation of gene regulatory mechanisms controlled by the extracytoplasmic function (ECF) sigma factors. Comparative analysis of the extracellular proteome of the wide type and a mutant for *rsuA*, which encodes a cognate anti-sigma factor of an ECF σ^U^, identified 79 extracellular proteins in the mutant, including abundant lipoproteins, proteases, phosphatases, and esterases. The involvement of these targets suggests that σ^U^ may play an important role in the stress response at the cell surface by triggering remodeling of cell envelope [90]. 

The mechanism of differentiation in streptomycetes has begun to be deduced by proteomics. Two related studies attempted to unveil the players that are involved in the transition from an early vegetative mycelium to a multinucleated mycelium stage. A novel technique named iTRAQ (isobaric tags for relative and absolute quantitation) labeling, coupled with LC-MS/MS were used for temporal profiling of proteins in the two stages, and spatial profiling of the membrane and cytosolic fractions of the proteomes [91]. Three hundred and fifty-nine (359) proteins were identified in at least one developmental stage. A large portion (83%) showed similar up- or down-regulation patterns with similar quantities in both liquid and solid cultures. More specifically, while the vegetative stage was characterized by abundant proteins for primary metabolism, the second reproductive stage involved up-regulation of proteins important for antibiotic production, for example, the biosynthesis of actinorhodin and type II polyketide [91].

The previously under-studied phosphoproteome (the regulatory machinery of phosphorylation by serine/threonine protein kinases (STPKs) and tyrosine protein kinases (YPKs)) has recently, for the first time, been delineated in *S. coelicolor* by a combination of a phosphopeptide enrichment assay and a nano LC-MS/MS assay. The phosphoproteome included at least 40 putative phosphoproteins, whose functions range from regulation, signaling, primary metabolism, transport, protein biosynthesis , to cell division [92]. 

## METABOLOMICS

Metabolomics, the study of complement of metabolic capability of an organism, has come into its own in streptomycetes, which house a powerful and sophisticated machinery for secondary metabolism. The first genome-wide stoichiometric model for *S. coelicolor* was reconstructed *in silico* in 2005 [93]. A total of 971 reactions encompassed 819 metabolic conversions and 152 transport reactions and involved 500 metabolites. Flux-balance analysis (FBA) was used to model the network topology and dynamics. Alam *et al.* [94] combined the FBA and the analysis of time-series transcriptomes to monitor the metabolic flux and gene expression profiles. They identified a group of key proteins for metabolic switching from primary metabolism to secondary metabolism, including at least 15 genes with predicted functions for vitamin B12 (cobalamin) biosynthesis, 10 genes implicated in calcium-dependent antibiotic (CDA) biosynthesis, and three genes involved in ectoine biosynthesis. To date, the most densely sampled time-series analysis of streptomycetes was conducted by Nieselt *et al.* [95]. This study monitored the whole genome expression for consecutive 60 hours using a customized Affymetrix genechip. Gene clusters with synchronized expression over the time course were identified; their expression seemed to be coordinated with the phase transition. 

The metabolic profiles measured and determined by MS assays provide detailed maps of the metabolite structure and concentration under specific conditions. Recently, the global metabolome under salt stress conditions was revealed by liquid chromatography-mass spectrometry [96], using comparisons among the metablomes of a wild type, mutants with progressive knockouts of the ectoine biosynthesis pathway, and mutants for stress regulators (OsaB and σ^B^). Besides proline, the classical salt-responsive metabolite, other amino acids, such as arginine, phenylalanine, methionine, tryprophan, and (iso)leucine could provide potential osmoprotection. Characterization of the secondary metabolome in *S. coelicoflavus* was accomplished by ultraperformance liquid chromatography coupled with electrospray ionization mass spectrometry (UPLC/ESIMS) [97], leading to the discovery of 80 acarviostatin family analogs, which included 65 novel oligomer compounds. 

## TOWARD A SYSTEMS LEVEL UNDERSTANDING OF *STREPTOMYCES* BIOLOGY

An increasing body of evidence suggests that key features of streptomycetes, such as gene regulation, stress response, secondary metabolism, and morphological differentiation, are not stand-alone properties. To the contrary, they are somewhat associated with or related to each other; for example, the proteins that are involved in secondary metabolism can also be regulators for differentiation and stress response. Many of the studies surveyed herein focus on the delineation of components and interaction of cellular networks [98], representing significant steps toward a global view of *Streptomyces* biology. In its infancy, *Streptomyces* systems biology faces several major challenges. Some of these challenges stem from technical difficulties such as unsatisfactory sensitivity for detecting low-abundance proteins, unavailability of PCR-like large scale characterization techniques for proteins, and complexity due to various types of epigenetic regulation. Computational challenges also plague efforts in systems biology, ranging from the lack of data quality assurance, and a pressing need for effective strategies for integrating heterogeneous data, to the development of accurate, robust, and adaptable modeling frameworks [99]. The complex nature of *Streptomyces* biology calls for network models that meet the following criteria: (1) they should be less subjective and are useful in sorting out the dependency between system variables and in visualizing otherwise obscure relationships; (2) they should be able to deal with noise, error and uncertainty; (3) they should incorporate principles for integrating data; (4) they should be able to cope with sparse data; and (5) they should permit circular regulatory relationships and the use of continuous functions in the cellular networks. A marriage between bioinformatics/computational biology and *Streptomyces* -omics holds new promises to a better understanding of the structure, dynamics, design principles and control mechanisms of streptomycetes, leading to a network view that will allow us to transform disparate types of high throughput data into biological insights. 

## SUPPLEMENTARY MATERIAL

Supplementary material is available on the publishers Web site along with the published article.

## Figures and Tables

**Table 1.** The *Streptomyces* Genome Projects

Organism	Chromosome Size (Mb)	ORF	GC%	Ref Seq/GenBank Accession	Sequence Status	References
*S. avermitilis*	9.0	7580	70	NC_003155	Completed	[21]
*S. bingchenggensis*	11.9	10023	71	CP002047	Completed	[25]
*S. cf. griseus*	8.7	7265	72	NZ_ADFC00000000	Completed	[28]
*S. coelicolor*	8.7	7768	72	NC_003888	Completed	[2]
*S. flavogriseus*	7.3	6298	71	CP002475	Completed	
*S. griseus*	8.5	7136	72	NC_010572	Completed	[24]
*S. scabiei*	10.1	8746	71	NC_013929	Completed	[27]
*S. sp. *Tü6067	7.4	6466	73	NZ_AFHJ01000000	Completed	[26]
*S. venezuelae*	8.2	7453	72	FR845719	Completed	
*S. clavuligerus*	6.7	5463	72	NZ_ADWJ00000000	Draft	[51, 101]
*S. albus*	6.6	5902	72	NZ_ABYC00000000	Draft	
*S. ghanaensis*	8.2	7891	70	NZ_ABYA00000000	Draft	
*S. griseoflavus*	7.4	6338	68	NZ_ACFA00000000	Draft	
*S.griseoaurantiacus*	7.7	6839	72	NZ_AEYX00000000	Draft	[102]
*S. hygroscopicus*	10.5	9194	69	NZ_ACEX00000000	Draft	
*S. lividans*	8.2	7551	71	NZ_ACEY00000000	Draft	
*S. pristinaespiralis*	7.6	6869	68	NZ_ABJI00000000	Draft	
*S. roseosporus*	7.8	7056	71	NZ_ABYX00000000	Draft	
*S. *S4	7.5	N/A	N/A	CADY01000000	Draft	[103]
*S. sp. *AA4	9.2	8419	69	NZ_ACEV00000000	Draft	
*S. sp. *ACTE	7.4	6521	71	NZ_ADFD00000000	Draft	
*S. sp. *C	7.9	7540	70	NZ_ACEW00000000	Draft	
*S. sp.* e14	7.1	6195	68	NZ_ACUR00000000	Draft	
*S. sp. *Mg1	7.1	6975	70	NZ_ABJF00000000	Draft	
*S. sp. *PP-C42	9.6	N/A	72	AEWS00000000	Draft	[104]
*S. sp. *SA3_actF	7.2	6823	73	NZ_ADXB00000000	Draft	
*S. sp. *SA3_actG	7.4	6547	73	NZ_ADXA00000000	Draft	
*S. sp. *SPB74	6.5	5742	70	NZ_ABJG00000000	Draft	
*S. sp. *SPB78	6.9	6386	69	NZ_ACEU00000000	Draft	
*S. sviceus*	9.1	8205	69	NZ_ABJJ00000000	Draft	
*S. violaceusniger*	11.0	9485	70	NZ_AEDI00000000	Draft	
*S. viridochromogenes*	8.5	7714	70	NZ_ACEZ00000000	Draft	

## ABBREVIATIONS

2-D: = Two-dimensional
ABC: = ATP-binding cassette
Act: = Actinorhodin
CDA: = Calcium-dependent antibiotic
ChIP-chip: = Chromatin immunoprecipitation-on-chip
ECF: = Extracytoplasmic function
ESIMS: = Electrospray ionization mass spectrometry
FBA: = Flux-balance analysis
IJISS: = Ink-jet in situ synthesis
iTRAQ: = Isobaric tags for relative and absolute quantitation
LC: = Liquid chromatography
LSE: = Lineage-specific expansion
MALDI–TOF: = Matrix-assisted laser desorption ionization–time-of-flight
MS: = Mass spectrometry
MUDPIT: = Multidimensional protein identification technology
ORF: = Open reading frame
Red: = Undecylprodigiosin
PTM: = Post-translational modification
SARP: = *Streptomyces* antibiotic regulatory protein
STPK: = Serine/threonine protein kinase
Tat: = Twin-arginine translocation
TIR: = Terminal inversed repeat
TP: = Terminal protein
UPLC: = Ultraperformance liquid chromatography
VB: = Virginiae butanolides
YPK: = Tyrosine protein kinase
