# Supplementary material for: The -omics Era- Toward a Systems-Level Understanding of Streptomyces
Source: Curr Genomics. 2011 Sep;12(6):404–16. doi: 10.2174/138920211797248556 (PMC3178909; doi:10.2174/138920211797248556)
Supplement: Supplementary file 1 [file CG-12-404_SD1.pdf]

Current Cancer Drug Targets, 2011, Vol. 11, No. 8

SUPPORTIVE/SUPPLEMENTARY MATERIAL

Table S1. The Complete List of Primers Used for PCR Analysis

Gene name and symbol	*Accession number*	*Forward primer sequence*	*Reverse primer sequence*
solute carrier family 38, member 2 (SLC38A2, SNAT2)	NM_018976	5' ATG AAG AAG GCC GAA ATG GGA 3'	5' TGC TTG GTG GGG TAG GAG TAG 3'
cyclin-dependent kinase inhibitor 1A (CDKN1A, p21)	NM_000389	5' CCT GTC ACT GTC TTG TAC CCT 3'	5' GCG TTT GGA GTG GTA GAA ATC T 3'
DNA-damage-inducible transcript 3 (CHOP)	NM_004083	5' CTT CTC TGG CTT GGC TGA CT 3'	5' TCC CTT GGT CTT CCT CCT CT 3'
GLUL glutamate-ammonia ligase (GS)	NM_002065	5' TCA TCT TGC ATC GTG TGT GTG 3'	5' CTT CAG ACC ATT CTC CTC CCG 3'
ASNS asparagine synthetase (AS)	NM_001178075	5' GAT TGC CTT TCT GTT CAG TGT CT3'	5' GGG TCA ACT ACC GCC AAC C 3'
RPL15 ribosomal protein L15 (RPL15)	NM_002948	5' GCA GCC ATC AGG TAA GCC AAG 3'	5' AGCGGACCCTCAGAAGAAAGC 3'

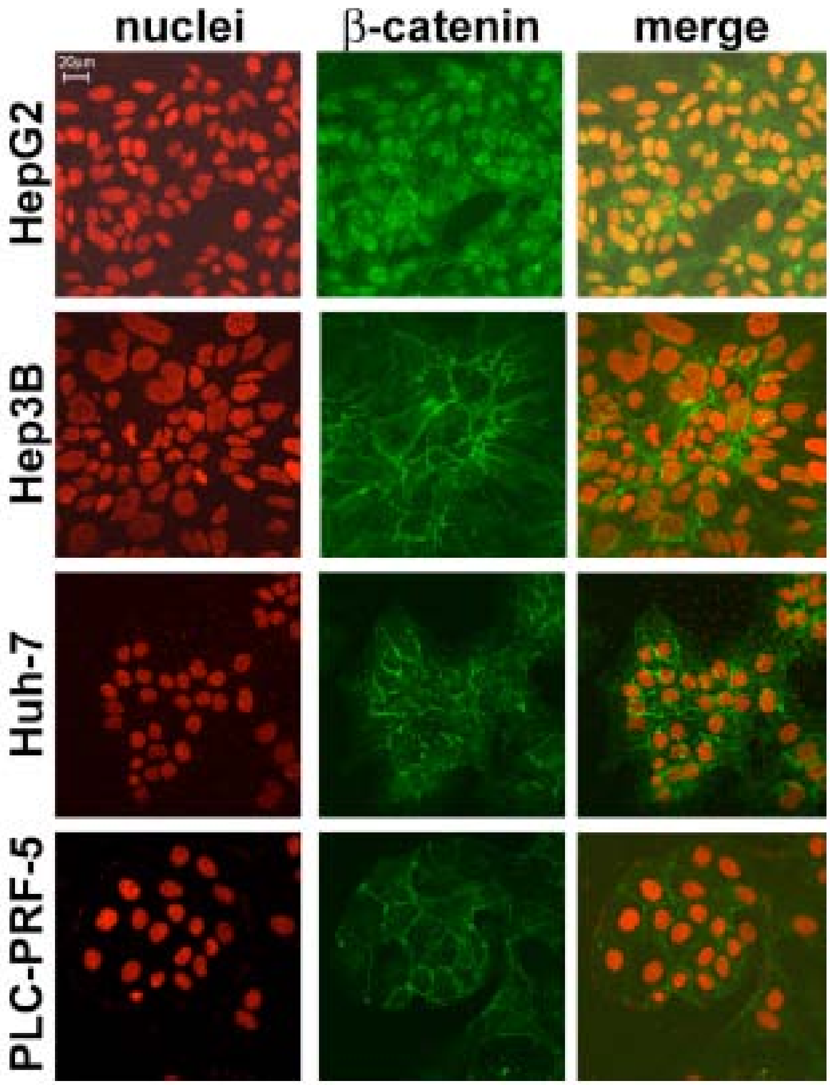

Figure S1. Merged images of propidium iodide labelling and β-catenin expression in HCC cells. The same images reported in Fig. 1 are shown in red scale (propidium iodide, nuclei), green scale (β-catenin) and as merged images. Note the evident colocalization (yellow) of red and green signals in HepG2 cells and the prevalent peripheral pattern of β-catenin in the other cell lines.

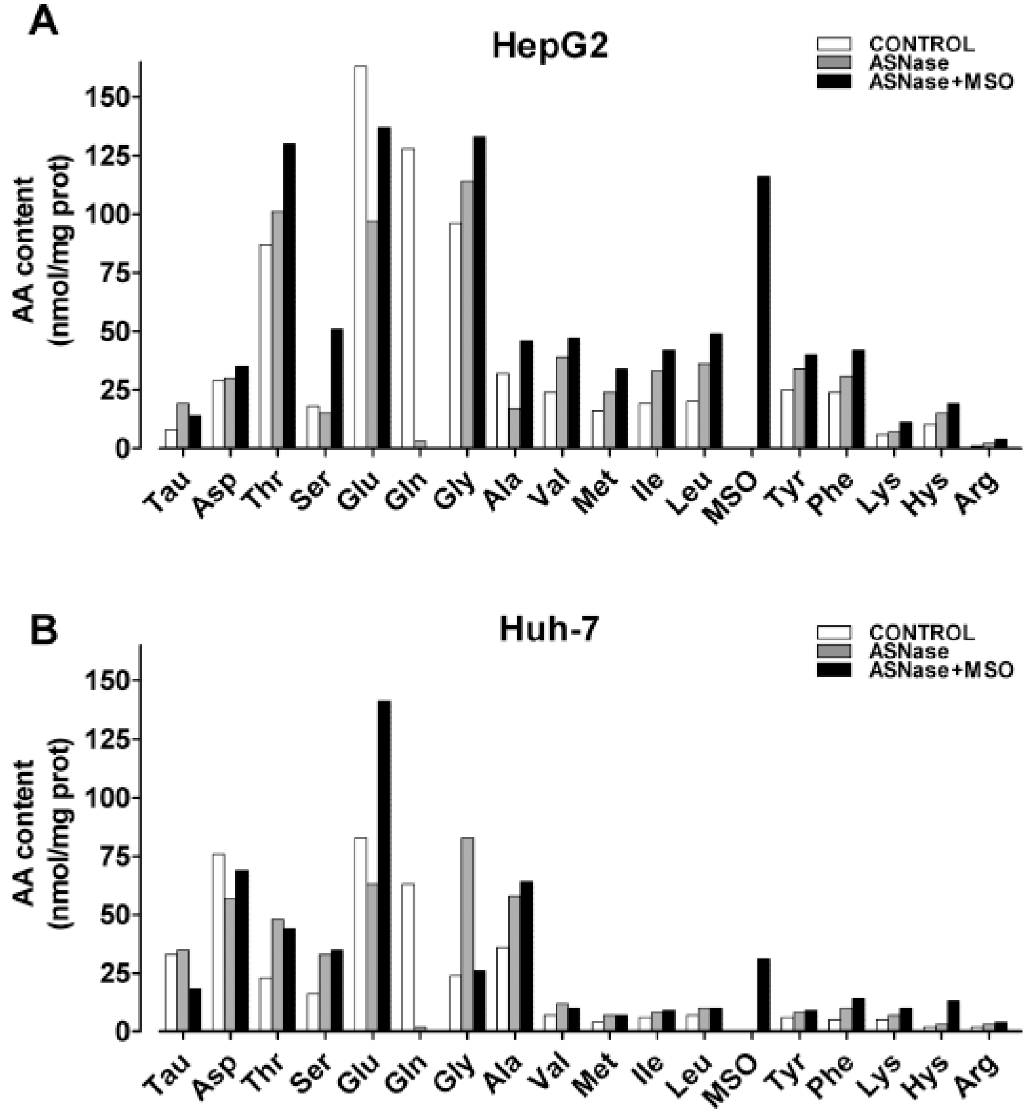

Fig. (S2). Amino acids in HepG2 (**A**) and Huh-7 (**B**) cells treated with ASNase and MSO. Cell contents of amino acids were determined, as described in Materials and Methods, in cells incubated for 24 hours in standard growth medium (Control) or in medium containing ASNase (1 U/ml) or ASNase (1 U/ml) + MSO (1 mM). A representative experiment, performed twice with comparable results, is shown.

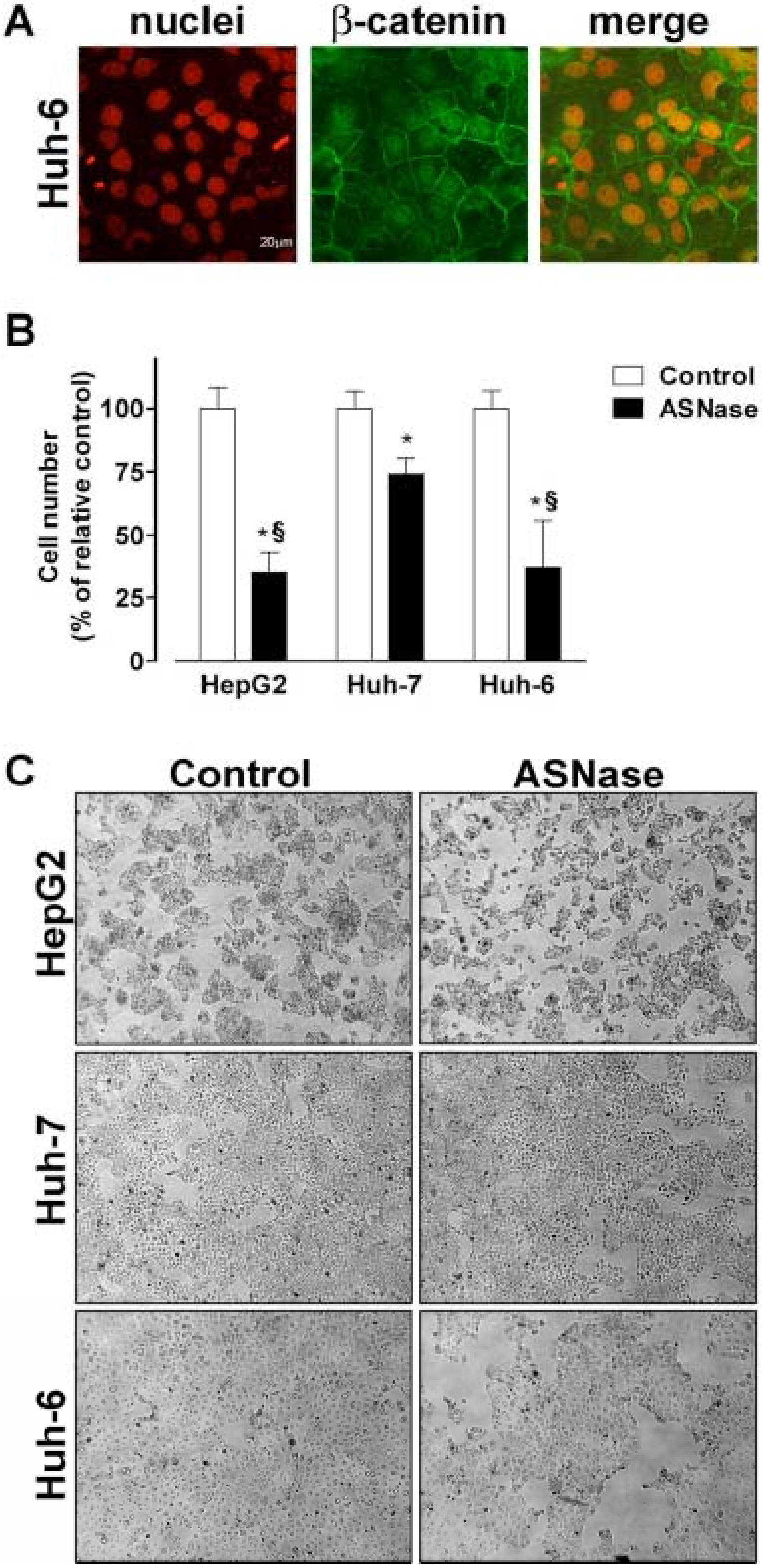

Fig. (S3). β-catenin expression and ASNase effects in Huh-6 cells. **A.** Immunofluorescence of β-catenin. The same representative field is shown in red scale (propidium iodide, nuclei), green scale (β- catenin) and as a merged image. Note the strong colocalization (yellow) although membrane associated distribution of β-catenin is also evident. Single confocal section. **B, C.** HepG2, Huh-7 and Huh-6 cells were treated with ASNase (1 U/ml) for 72h. at the end of the incubation cultures were photographed (original magnification 40x, C) and cells counted with a Coulter Z1 particle counter (B). For B, data are means of three determinations with SD shown. *, § $p < 0.05$ *vs*. control, untreated cultures or Huh-7 cells treated with ASNase, respectively.
